# Ferroptosis and Its Potential Role in Human Diseases

**DOI:** 10.3389/fphar.2020.00239

**Published:** 2020-03-17

**Authors:** Chu Han, Yuanyuan Liu, Rongji Dai, Nafissa Ismail, Weijun Su, Bo Li

**Affiliations:** ^1^School of Chemistry and Chemical Engineering, Beijing Institute of Technology, Beijing, China; ^2^Advanced Research Institute of Multidisciplinary Science, Beijing Institute of Technology, Beijing, China; ^3^Beijing Key Laboratory for Separation and Analysis in Biomedicine and Pharmaceuticals, School of Life Science, Beijing Institute of Technology, Beijing, China; ^4^Neuroimmunology, Stress and Endocrinology (NISE) Lab, School of Psychology, Faculty of Social Science, University of Ottawa, Ottawa, ON, Canada; ^5^Brain and Mind Research Institute, University of Ottawa, Ottawa, ON, Canada; ^6^School of Medicine, Nankai University, Tianjin, China

**Keywords:** ferroptosis, signaling pathways, reactive oxygen species, pharmacology design, degenerative diseases

## Abstract

Ferroptosis is a novel regulated cell death pattern discovered when studying the mechanism of erastin-killing RAS mutant tumor cells in 2012. It is an iron-dependent programmed cell death pathway mainly caused by an increased redox imbalance but with distinct biological and morphology characteristics when compared to other known cell death patterns. Ferroptosis is associated with various diseases including acute kidney injury, cancer, and cardiovascular, neurodegenerative, and hepatic diseases. Moreover, activation or inhibition of ferroptosis using a variety of ferroptosis initiators and inhibitors can modulate disease progression in animal models. In this review, we provide a comprehensive analysis of the characteristics of ferroptosis, its initiators and inhibitors, and the potential role of its main metabolic pathways in the treatment and prevention of various diseased states. We end the review with the current knowledge gaps in this area to provide direction for future research on ferroptosis.

## What Is Ferroptosis?

### Definition and Discovery of Ferroptosis

Biological growth and the onset of disease are closely linked to cell death. There are two main patterns of cell death: 1) accidental cell death (ACD), and 2) regulated cell death (RCD). One type of RCD is programmed cell death or apoptosis, which occurs normally throughout development (Galluzzi et al., 2015). Apoptosis was in fact the first RCD mechanism discovered, and since then other RCD patterns have also been identified such as necrosis, pyroptosis, and, more recently, ferroptosis (Kroemer et al., 2009).

Studies on ferroptosis initiators and related mechanisms emerged several decades ago, prior to the establishment of the term “ferroptosis”. As early as 1989, Murphy's group discovered that glutamate caused neuronal cell death by inhibiting system x_c_^-^ (Murphy et al., 1989), which was later named “oxytosis” by Maher's group in 2001 (Tan et al., 2001). Recent studies found that ferroptosis and oxytosis had several common characteristics, such as the role of lipoxygenase, ROS production, and gene expression (Lewerenz et al., 2018). However, there are discrepancies in a few protein-signaling pathways between ferroptosis and oxytosis (Neitemeier et al., 2017). In 2008, two compounds, ras-selective lethal small molecules 3 (RSL3) and 5 (RSL5), were screened using a high-throughput method that could selectively induce cell death in cells carrying RAS mutant subtype genes (Yang and Stockwell, 2008). The results facilitated the identification of the lethal mechanism of the compound erastin (Dolma et al., 2003). Erastin was discovered to induce cell death without nuclear morphological changes, DNA fragmentation, and caspase3 activation, and this process could not be reversed by caspase inhibitors. Therefore, it is a regulated but non-apoptotic form of cell death (Dixon et al., 2012). This erastin-induced cell death pattern is accompanied by an increasing concentration of lipid hydroperoxides (Yagoda et al., 2007) and is inhibited by iron chelators (e.g., deferoxamine mesylate) (Yang and Stockwell, 2008).

In 2012, Stockwell's group was the first to report and name this iron-dependent cell death pattern characterized by increased lipid reactive oxygen species (ROS) as “ferroptosis” (Dixon et al., 2012). In 2018, the Nomenclature Committee on Cell Death (NCCD) defined ferroptosis as “a form of regulatory cell death initiated by oxidative perturbations of the intracellular microenvironment that is under constitutive control by glutathione peroxidase 4 (GPX4) and can be inhibited by iron chelators and lipophilic antioxidants” (Galluzzi et al., 2018).

### Characteristics of Ferroptosis

Ferroptosis differs from apoptosis, necrosis, and pyroptosis in morphological and physiological characteristics (**Table 1**) (Yagoda et al., 2007; Kroemer et al., 2009; Vandenabeele et al., 2010; Galluzzi et al., 2012; Aachoui et al., 2013; Kayagaki et al., 2015; Shi et al., 2015; Vande Walle and Lamkanfi, 2016; Wang et al., 2017; Galluzzi et al., 2018). For example, during ferroptosis, the nuclei of the cell remain intact (Friedmann Angeli et al., 2014), the chromatin is not aggregated (Xie et al., 2016a), and the plasma membrane is not broken or foamed. The shrinking mitochondria show greater inner membrane density while the outer membrane ruptures. In contrast to ferroptosis, apoptosis is characterized by cytoplasmic contraction, nuclear division, chromatin condensation, chromosomal DNA division (Galluzzi et al., 2012), and mitochondrial cytochrome c release (Galluzzi et al., 2018). In apoptotic cells, the plasma membrane foams and eventually forms a distinct intact vesicle (commonly referred to as an apoptotic body). On the other hand, mitochondrial permeability transition (MTP)-driven necrosis is characterized by cytoplasmic granulation, swelling of organelles and cells, loss of cell membrane integrity, and, ultimately, leakage of cellular content (Vandenabeele et al., 2010). In pyroptotic cells, early membrane rupture exposes the inner leaves of the plasma membrane to the extracellular surface, allowing cellular protein annexin V to bind to phosphatidylserine (PS) in the inner leaves. Pyroptotic cells are accompanied by the formation of caspase-1 activity-dependent 12-nm pores in the plasma membrane, leading to a flux of transmembrane ions, swelling of the cytoplasm, and, ultimately, the osmotic dissolution of the cell (Vande Walle and Lamkanfi, 2016). Thus, there are several key distinctions between ferroptosis and other cell death types.

**Table 1:** Comparison of Characteristics of Apoptosis, Necrosis, Pyroptosis and Ferroptosis.

	Definitions	Morphological features	Biochemical features
**Apoptosis**	Type of RCD initiated by perturbation of the extracellular or intracellular microenvironment; Demarcated by mitochondrial outer membrane permeabilization (MOMP); Precipitated by executioner caspases, mainly caspase3 (CASP3).	Rounding-up of the cell; Retraction of pseudopods; Reduction of cellular and nuclear volume (pyknosis); Nuclear fragmentation (karyorrhexis); Minor modification of cytoplasmic organelles; Plasma membrane blebbing; Engulfment by resident phagocytes *in vivo*.	Release of mitochondrial intermembrane space ‘IMS’ proteins; Respiratory chain inhibition.
**MTP-driven Necrosis**	Specific form of RCD triggered by perturbations of the intracellular microenvironment and relying on cyclophilin D (CYPD).	Rupture of plasma Membrane; Cytoplasm: cytoplasmic swelling. (Oncosis): Swelling of cytoplasmic organelles; Moderate chromatin condensation.	Caspase inhibition; NADPH oxidase activation; Neutrophil extracellular traps (NETs) release (in some instances).
**Pyroptosis**	A type of RCD that critically depends on the formation of plasma membrane pores by members of the gasdermin protein family; Often (but not always) as a consequence of inflammatory caspase activation.	The early membrane rupture of pyroptotic cells exposes the inner leaflet of the plasma membrane to the extracellular surface; Transmembrane ion fluxes; Cytoplasmic swelling; Osmotic lysis of the cell.	Caspase-1 activation; Caspase-7 activation; Secretion of IL-1β and IL18.
**Ferroptosis**	A form of RCD initiated by oxidative perturbations of the intracellular microenvironment that is under constitutive control by GPX4 and can be inhibited by iron chelators and lipophilic antioxidants.	Normal nuclei and shrinking mitochondria that show increased membrane density and outer mitochondrial membrane rupture.	Iron and ROS accumulation; Inhibition of system x_c_^−;^ With decreased cystine uptake; GSH depletion and increased; NAPDH oxidation Release of arachidonic acid mediators.

The most important biochemical features of ferroptosis are the elevated levels of lipid hydroperoxides (LOOH) and ferrous ion (Fe^2+^) concentration, as ferroptotic cells produce excessive reactive oxygen species, which initiates lipid peroxidation *via* Fenton Chemistry. Glutathione peroxidase 4 (GPX4), an enzyme that specifically reduces lipid peroxide to the corresponding alcohol, is another central regulator of ferroptosis (Yang et al., 2014). In addition, glutathione (GSH) acts as a GPX4 cofactor and maintains the level of GPX4 through the exchange of glutamate and cystine *via* the antiporter system x_c_^-^ (Stockwell et al., 2017).

The genes that control ferroptosis also differ from those that control other forms of cell death. Six protein encoding genes necessary for ferroptosis were screened in HT1080 and Calu-1 cells using shRNA library targeting genes encoding predicted mitochondrial proteins, including genes encoding ribosomal protein L8 (RPL8), iron response element binding protein 2(IREB2), ATP synthase F_0_ complex subunit C3 (ATP5G3), citrate synthase (CS), tetratricopeptide repeat domain 35 (TTC35), and acyl-CoA synthetase family member 2 (ACSF2) proteins. In addition, TFRC, ISCU, FTH1, and FTL are key genes in ferroptosis that control iron uptake, metabolism, and storage by affecting Fe^2+^ levels (Dixon et al., 2012).

These genes are different from the ones that control apoptosis (e.g. BH3 interacting domain death agonist (BID), BCL2 antagonist/killer 1(BAK1), BCL2 associated X (BAX), apoptosis inducing factor mitochondria associated 1(AIFM1)) or genes that control other cell death patterns (e.g. genes peptidylprolyl isomerase F (PPIF) involved in MPT-driven necrosis) (Dixon et al., 2012; Galluzzi et al., 2018).

## Regulatory Mechanisms of Ferroptosis

### Lipid Oxidation Metabolism

**Figure 1:**
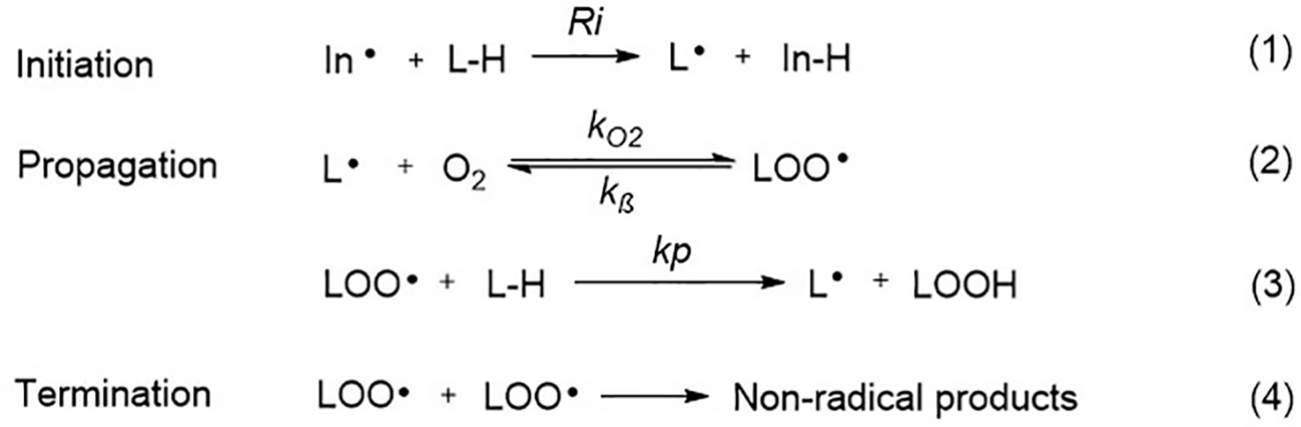
The free radical chain mechanism of lipid peroxidation.

Ferroptosis is linked to a fatal accumulation of lipid peroxidation, which is the archetype free radical chain reaction formally resulting in the insertion of O_2_ into a C-H bond in the oxidizable free polyunsaturated fatty acids (PUFAs) (**Figure 1** **Eq. 1.1-1.4**). This leads to the formation and accumulation of LOOH and ROS and causes ferroptosis. Any radical that can abstract an H-atom from an oxidizable substrate like PUFAs (L-H, **Figure 1** **Eq. 1.1**) can initiate the lipid peroxidation process *in vivo*. The resultant carbon-centered alkyl radical (L**·**) reacts with molecular O_2_ in the environment at (or near) the rate of diffusion, giving rise to a peroxyl radical (LOO**·**). (**Figure 1** **Eq. 1.2**) (Maillard et al., 1983). The produced peroxyl radicals propagate the chain reaction at propagation rate *k*_p_ by abstracting H atom from another molecule of oxidizable substrate (L-H) to yield LOOH and a new alkyl radical (L**·**) (**Figure 1** **Eq. 1.3**). Radical-radical reactions are the final step resulting in non-radical products (**Figure 1** **Eq. 1.4**) (Russell, 1956).

The abundance and location of intracellular oxidizable substrates of lipid peroxidation determines the extent of lipid peroxidation and the extent of ferroptosis. Free polyunsaturated fatty acids are esterified into membrane phospholipids (Kagan et al., 2017) in the lipid metabolic process. Lipidomic analyses indicate that phosphatidylethanolamines (PEs) containing arachidonic acid (AA) or adrenic acid (AdA) are key membrane phospholipids and are further oxidized to phospholipid hydroperoxides (PE-AA/AdA-OOH) by non-enzymatic processes, such as the above mentioned free radical lipid peroxidation or Fenton chemistry drives ferroptosis (Doll et al., 2017; Kagan et al., 2017).

**Figure 2:**
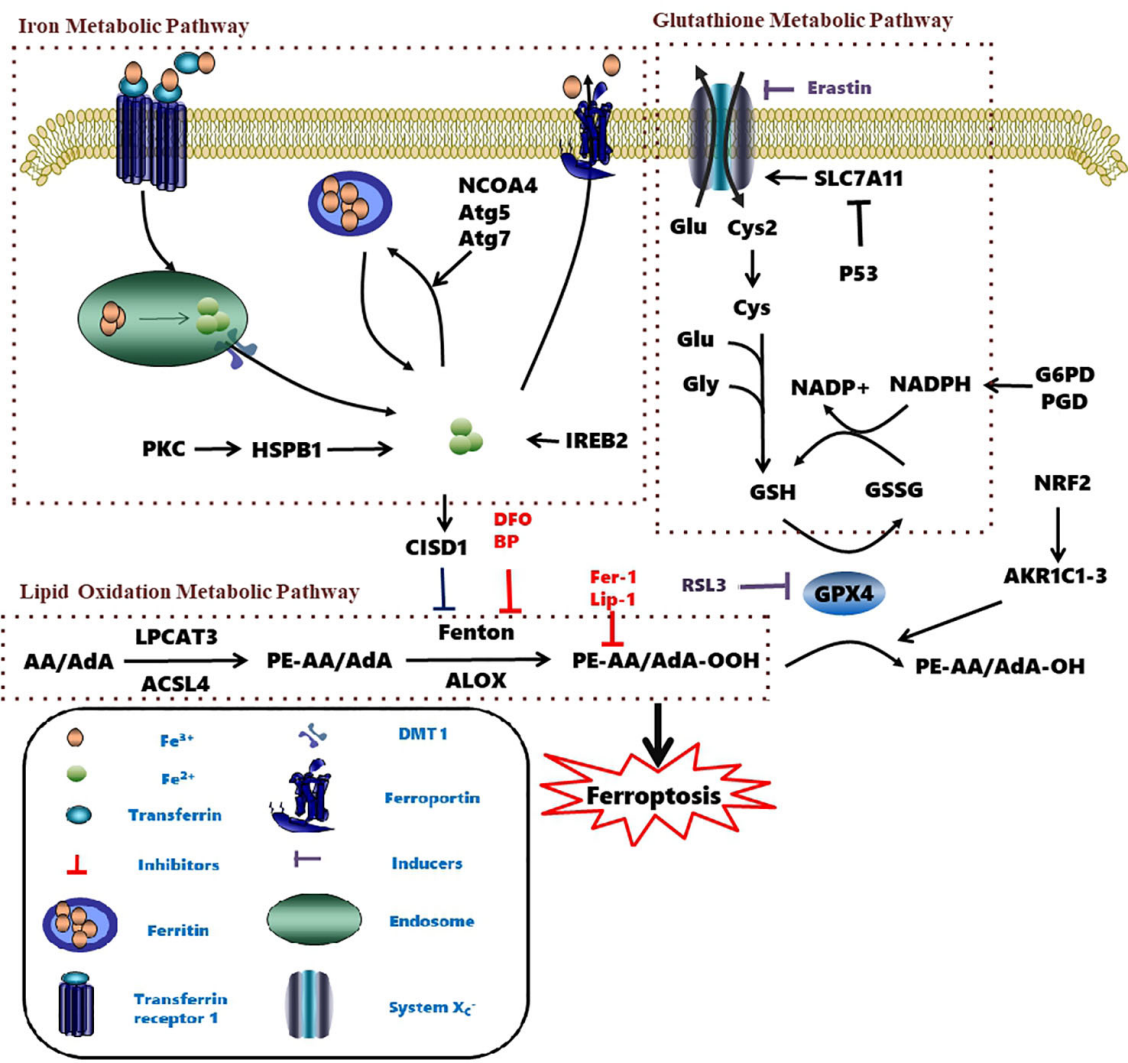
Metabolic pathways affecting ferroptosis. The brown box contains three currently known pathways: Lipid Oxidation Metabolism、Glutathione Metabolic Pathway、Iron Metabolic Pathway and some related mechanisms of action. Several pharmacological inducer have been shown to induce ferroptosis (eg erastin、RSL3). A variety of ferroptosis inhibitors inhibit iron death from various pathways (eg Fer-1、lip-1、BP、DFO). AA, Arachidonic acid; ACSL4, Acyl-CoA synthetase long-chain family member 4; AKR1C1-3, Aldo-keto reductase family 1 member C1-3; Atg5, autophagy-related 5; Atg7, autophagy-related 7; CISD1, CDGSH iron domain 1; Cys, cysteine; Cys2, cystine; DFO, Deferoxamine; DMT1, Divalent metal transporter 1; Fer-1, Ferrostatin-1; Glu, Glutamate; Gly, glycine; GPX4, Glutathione peroxidase 4; G6PD, Glucose-6-phosphate dehydrogenase; GSH, Glutathione; GSSH, Glutathione disulfide; IREB2, Iron-responsive element-binding protein 2; Lip-1, Liproxstatin-1; LOX, Lipoxygenase; LPCAT3, Lysophosphatidylcholine acyltransferase 3; NADPH, Nicotinamide adenine dinucleotide phosphate; NRF2, Nuclear factor erythroid 2-related factor 2; PE, Phosphatidylethanolamine; PGD, 6-Phosphogluconate dehydrogenase; PKC, Protein kinase C; RSL3, Ras-selective lethal small molecules 3; SLC7A11, Solute carrier family 7 member 11.

Two enzymes related to ferroptotic lipid metabolism were found through the haploid gene screening of chronic myeloid leukemia cell line KBM7 cells: acyl-CoA synthetase (a long-chain family member 4 (ACSL4) for synthesizing Pes), and lysophosphatidylcholine acyltransferase 3 (LPCAT3) for lipid remodeling. AA/AdA is acylated into membrane phospholipids by LPCAT3 and ACSL4 (PE-AA/AdA) (**Figure 2**) (Dixon et al., 2015; Kagan et al., 2017). The blocking of ACSL4 results in the suppression of AA or AdA esterification into PE, which reduces the sensitivity of mouse embryonic fibroblasts Pfa1 cells to ferroptosis (Kagan et al., 2017). In DU-145 cancer cells, overexpression of multiple AKR1C family members has been shown to up-regulate the aldo-keto reductase family 1 member C1-3 genes (including AKR1C1-3) and blocks ferroptosis. AKR1C1-3 encodes an aldehyde ketone reductase to reduce the final product of lipid peroxide (PE-AA/AdA-OOH) to the non-toxic corresponding lipid derived alcohol (PE-AA/AdA-OH) (Dixon et al., 2014). Together, these studies demonstrate that lipid peroxidation is a key factor in ferroptosis.

PUFAs could also be oxidized to the corresponding phospholipid hydroperoxides by lipoxygenases (LOXs) (Kagan et al., 2017; Raefsky et al., 2018). However, whether LOX isozymes are essential for ferroptosis remains controversial. While research using a mouse model of GPX4-induced acute renal failure showed that LOX15 is not required for ferroptosis (Friedmann Angeli et al., 2014), work on a variety of damaged cells shows that phosphatidylethanolamine binding protein 1 (PEBP1) forms a complex with ALOX15 and acts as a scaffold protein to positively regulate ferroptosis by (Wenzel et al., 2017). In addition, it was found that deuterated AA with deurerium at the 7-hydrogen atom position of bisallyl was not oxidized to reduce the sensitivity of cells to ferroptosis. These results proved that AA was involved in the lipid peroxidation during ferroptosis (Raefsky et al., 2018).

### Glutathione Metabolic Pathway

The synthesis of tripeptide glutathione (GSH) appears to protect cells from ferroptotic death. The functional activity of glutathione peroxidase 4 (GPX4) is dependent on the biosynthesis of GSH (Yang et al., 2014). More specifically, depletion of GSH causes GPX4 inactivation and increases intracellular lipid peroxidation, resulting in ferroptosis (Cao and Dixon, 2016; Shimada and Stockwell, 2016).

System x_c_^-^ also regulates ferroptosis (Yang and Stockwell, 2008). System xc- consists of a 12-pass transmembrane protein transporter solute carrier family 7 member 11 (SLC7A11) and a single-pass transmembrane regulatory protein solute carrier family 3 member 2 (SLC3A2). Glutamate and cystine are exchanged at a 1:1 ratio by system x_c_^-^ (Sato et al., 1999). Tripeptide GSH is synthesized in two steps from cysteine, glutamic acid, and glycine (Yang et al., 2016; Stockwell et al., 2017). The efficiency of this synthetic process is limited to the concentration of cysteine in the substrate. Inhibition of systemic x_c_^-^ depletes intracellular cysteine, leads to a decrease in glutathione concentration and triggers oxidative stress, and increases the sensitivity of cells to ferroptosis (Cao and Dixon, 2016). However, upregulation of SLC7A11 inhibits erastin-induced ferroptosis (Dixon et al., 2012; Shimada and Stockwell, 2016).

Moreover, some mammals use methionine as a sulfur donor to synthesize new cysteines *via* the trans-sulfuration pathway. Although mammals usually rely solely on extracellular uptake as the major source of cysteine, the trans-sulfuration pathway acts as a compensatory source of cysteine when system x_c_^-^ uptake is inhibited (Shimada and Stockwell, 2016). A genome-wide siRNA screening of erastin-induced ferroptosis inhibitors showed that down-regulation of cysteinyl-tRNA synthase (CARS) leads to an up-regulation of the trans-sulfuration pathway and an inhibition of erastin-induced ferroptosis. This result supports the hypothesis that the trans-sulfuration pathway is a regulator of ferroptosis that compensates for cysteine depletion induced by cysteine update inhibition (Hayano et al., 2016).

### Iron Metabolic Pathway

The homeostasis of intracellular iron is dependent on the balance between iron absorption, output, utilization, and storage (Galaris et al., 2019). Ferric iron (Fe^3+^) enters the endosome through the membrane protein transferrin receptor 1 (TFR1) and it is reduced to ferrous iron (Fe^2+^) by iron reductase. The unstable Fe^2+^ is then released into the labile iron pool in the cytoplasm by the divalent metal transporter 1 (DMT1). Excess iron ions are either stored in ferritin heteropolymers in the form of Fe^3+^ or are released extracellularly *via* the membrane protein ferroportin.

**Figure 3:**
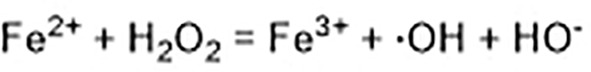
Fenton reaction.

Excessive ferrous iron provides electron-promoting lipid peroxidation through the Fenton reaction (**Figure 3**) and produces ROS, which triggers ferroptosis. Many autophagy-related genes can also activate ferroptosis. Inhibition of autophagy-related 5 7 genes reduce the accumulation of free iron and inhibit ferroptosis (Gao et al., 2016). Down-regulation of nuclear receptor coactivator 4 (NCOA4), a ferritin phagosome receptor, also inhibits ferritin phagocytosis and reduces Fe^2+^ content in cells (Gao et al., 2016; Hou et al., 2016). Iron-responsive element-binding protein 2 (IREB2) encodes a major regulator of iron metabolism, and studies have shown that shRNA-mediated silencing of IREB2 alters iron uptake, metabolism, and storage-related genes like TFRC, ISCU, FTH1, and FTL expression (Dixon et al., 2012). Heat shock protein beta-1 (HSPB1) (Sun et al., 2015) and CDGSH iron domain 1 (CISD1) (Yuan et al., 2016) also affect iron metabolism and regulate ferroptosis. In Hela cells, activation of HSPB1 phosphorylation using protein kinase C (PKC) reduces iron levels and blocks ferroptosis (Sun et al., 2015). CISD1, located in the outer membrane of mitochondria, inhibits the uptake of iron ions by mitochondria and also blocks ferroptosis (Yuan et al., 2016). However, oncogenic RAS increases iron content in cells, upregulates TFR, and downregulates ferritin (Yang and Stockwell, 2008). The RAS–RAF–MEK pathway sensitizes cancer cell lines with RAS to ferroptosis *via* mitochondrial voltage-dependent anion channels 2/3 (VDAC2/3) (Yagoda et al., 2007). In addition, tubulin negatively regulates mitochondrial metabolism by turning off VDAC.

**Figure 4:**
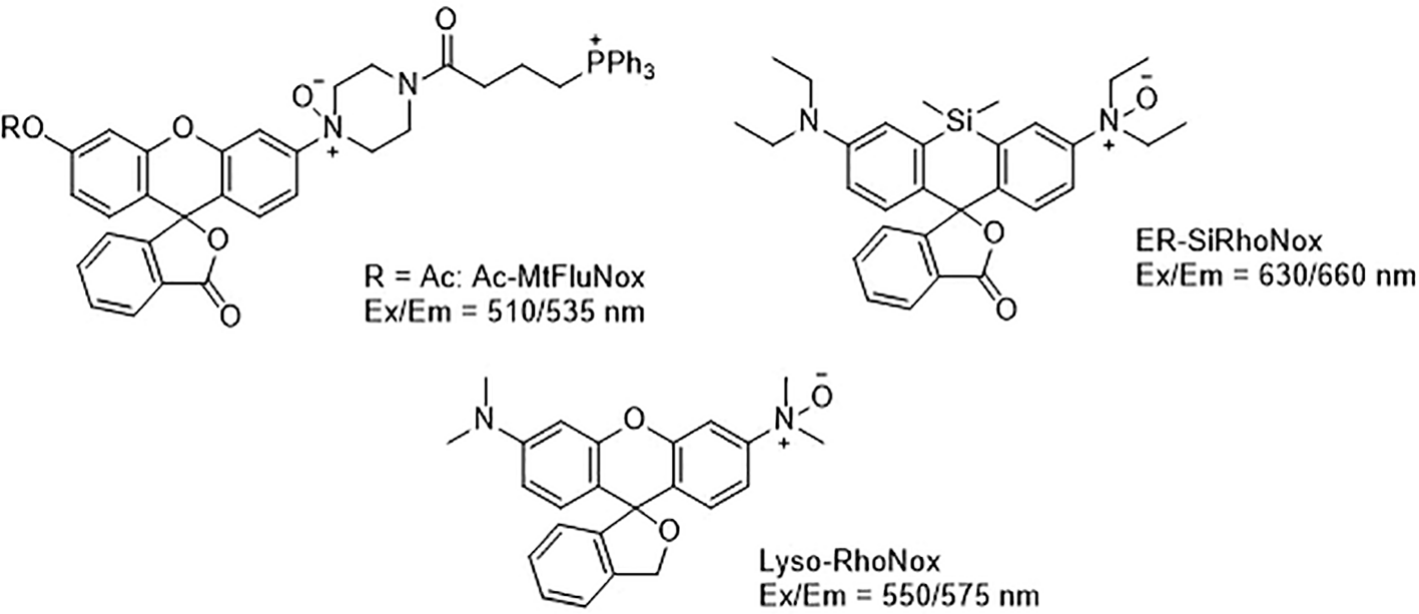
Structures of Ac-MtFluNox, Lyso-RhoNox, and ER-SiRhoNox.

Developing novel fluorescent probes which could detect labile Fe^2+^ levels in various cells or organs in live animals would definitely help further understanding of the role of iron in ferroptosis under various conditions. For example, an abnormal increase in Fe^2+^ in lysosomes and endoplasmic reticulum has been observed before the death of HT1080 cells induced by erastin using a variety of novel fluorescent probes (**Figure 4**) (Hirayama et al., 2019). This study further shows that iron metabolism plays a key role in the ferroptosis process (Yang and Stockwell, 2008).

### Other Related Signaling Metabolism

In addition to the above-mentioned signaling pathways, nicotinamide adenine dinucleotide phosphate (NADPH) also impacts ferroptosis sensitivity. NADPH is one of the most important reducing agents in cells and protects from excess oxidative damage. In fact, knocking out NADPH increases the sensitivity of fibrosarcoma HT1080 cells to ferroptosis (Shimada et al., 2016a). In the presence of NADPH, glutathione reductase reduces cystine-glutathione disulfide (GSSG) to GSH and increases ferroptosis sensitivity in many cancer cell lines (Zhao et al., 2017).

The nuclear factor erythroid 2-related factor 2 (Nrf2) is a key regulator of the antioxidant response (Sun et al., 2016). Under normoxic cellular conditions, Nrf2 is bound by Kelch-like ECH-associated protein 1 (Keap1) and persists in an inactivated status through ubiquitination and degradation in the proteasome (Reisman et al., 2009). Upon oxidative or electrophilic stress, Nrf2 becomes unleashed from the Keap1 protein binding and translocates to the nucleus (Fan et al., 2017). In the nucleus, Nrf2 transcripts antioxidant responsive element (ARE)-dependent genes in order to balance oxidative mediators and maintain cellular redox homeostasis (Hayes and Dinkova-Kostova, 2014). The above-described process is also regulated by autophagy. The autophagy receptor p62 is a multifunctional protein located throughout the cell which could activate Nrf2 through inactivation of Keap1 (Komatsu et al., 2010). In 2016, Tang's group discovered that the p62-Keap1-Nrf2 antioxidative signaling pathway was involved in the ferroptosis inhibition in HCC cells (Sun et al., 2016). In addition, it had been found that the Nrf2-mediated anti-ferroptosis activity was dependent on the induction of NQO1, heme oxygenase-1 (HO-1), and ferritin heavy chain (FTH1) (Sun et al., 2016). These findings also provide a potential molecular link between ferroptosis and autophagy in the hepatocellular carcinoma cells.

With the continuous research progress on ferroptosis, research evidence has indicated that the occurrence of ferroptosis crosstalk with autophagy (Hou et al., 2016; Kang and Tang, 2017) through pathways other than p62-Keap1-Nrf2. Tang's group demonstrated that knockout or knockdown of autophagy-related 5 (Atg5) and autophagy-related 7 (Atg7) degraded ferritin in fibroblasts and cancer cells, resulting in the limitation of erastin-induced ferroptosis by decreasing intracellular ferrous iron levels and lipid peroxidation (Hou et al., 2016). Other studies also confirmed that signaling pathways closely related to the occurrence of autophagy such as GPX4, SLC7A11, Nrf2, p53, HSPB1, and ACSL4 were also involved in ferroptosis (Kang and Tang, 2017). Torii's group found that autophagy contributed to ferroptosis through the generation of lysosomal ROS in the N-Ras-mutated HT1080 cells (Torii et al., 2016). In addition, they also found that autophagy and lysosomal activity inhibitors including Baf A_1_, PepA-Me, and ammonium chloride were effective in the prevention of erastin-induced ferroptosis (Torii et al., 2016; Sun et al., 2018). However, the occurrence of ferroptosis is not always related to autophagy, and the relationship between ferroptosis and autophagy or other cell death pathways needs to be further clarified in future research.

P53 also mediates cell cycle inhibition, senescence, and apoptosis, and contributes to the development of tumors (Jiang et al., 2015a). P53 regulates cellular metabolism through the SLC7A11 gene (Jiang et al., 2015b). More specifically, P53 inhibits system x_c_^-^ by down-regulating SLC7A11. Exposure to ROS in P53 gene-silencing human lung cancer H1299 cells does not change cell viability. However, exposure to ROS after activation of the P53 gene increases the cell death rate to 90%. This study shows that the antioxidant capacity is significantly reduced after activation of the P53 gene, and the cell death rate decreases by about 40-fold after the addition of the ferroptosis-specific inhibitor ferrostatin-1 (Jiang et al., 2015b). Therefore, it indicates that P53 plays an important role in the cellular ferroptotic-related ROS metabolic pathway.

It has been revealed that inhibition of cystine–glutamate exchange leads to the activation of endoplasmic reticulum (ER) stress response and upregulation of glutathione-specific gamma-glutamylcyclotransferase 1 (CHAC1) gene, therefore contributing to the glutathione degradation and ferroptosis execution (Rahmani et al., 2007; Dixon et al., 2014). Current research has indicated that ER stress and p53 upregulated modulator of apoptosis (PUMA) overexpression caused by ferroptosis inducers sets up an interaction between ferroptosis and apoptosis (Hong et al., 2017). It has been found that ferroptosis initiator artesunate (ART) could induce ER stress and elevate the expression of the pro-apoptotic PUMA through the ER stress–mediated PERK–eIF2a–ATF4–CHOP pathway but without inducing apoptosis (Hong et al., 2017; Lee et al., 2018). These findings provide new thought on the relationship between ferroptosis and apoptosis.

The ferroptosis suppressor protein 1 (FSP1) (formerly known as AIFM2) has recently been found to be independent of GSH and it could inhibit the transmission of lipid peroxides by reducing coenzyme Q10 to inhibit ferroptosis (Bersuker et al., 2019; Doll et al., 2019). Furthermore, *N-*myristoylation of FSP1 was found to be essential for ferroptosis inhibition and could provide a new target for the development of drugs targeting the inhibition of ferroptosis.

It has recently been discovered that intercellular interaction dictates ferroptosis. Epithelial extracellular E-cadherin regulates ferroptosis by intracellular NF2–YAP signaling. Antagonism of NF2 causes YAP to promote ferroptosis by up-regulating the associated signaling factors of ferroptosis, ACSL4, and TFRC. Intercellular E-cadherin interactions activate NF2 to inhibit ferroptosis in cells (Wu et al., 2019). However, whether other intercellular interactions dictate ferroptosis in other cell lines remains to be studied.

Currently, whether mitochondria are essential for ferroptosis remains controversial (Bock and Tait, 2019). On one hand, Stockwell's group found that cells lacking mitochondrial DNA were still sensitive to ferroptosis (Gaschler et al., 2018b). Therefore, their conclusion was that ROS generated by the mitochondrial electron transport chain was not essential to ferroptosis initiation. On the other hand, Jiang's group in 2019 showed that mitochondria did not play a role in GPX4 inhibition-induced ferroptosis, but it did play a crucial pro-active role in cysteine-deprivation-induced ferroptosis (Gao et al., 2019). In addition, Tang's group observed that ferroptosis initiator erastin induced the production of mitochondrial ROS (Yuan et al., 2016). They also demonstrated that intra-mitochondrial iron-mediated lipid peroxidation contributed to ferroptosis and CDGSH iron sulfur domain 1 (CISD1), an iron-containing outer mitochondrial membrane protein which limited mitochondrial iron uptake and therefore suppressed ferroptosis (Yuan et al., 2016). Based on the current research, the controversy on the role of mitochondria in ferroptosis remained to be solved.

## Initiators of Ferroptosis

Diseases like cancer can be treated by regulating cell death. In some pathological processes, treatment also works by depleting the essential cytokines which then results in cell death. Numerous studies have focused on discovering novel ferroptosis inducers to treat cancer (Hassannia et al., 2019). Ferroptosis inducers can be roughly classified into three categories: (1) system x_c_^-^ inhibitors, (2) GPX4 inhibitors, and (3) compounds that indirectly inhibit GPX4 activity by GSH depletion (**Table 2**).

**Table 2:** Initiators of Ferroptosis.

Compound	Target	Chemical structures	Model
Erastin (Dolma et al., 2003; Yagoda et al., 2007)	System x_c_^-^ and VDAC2/3	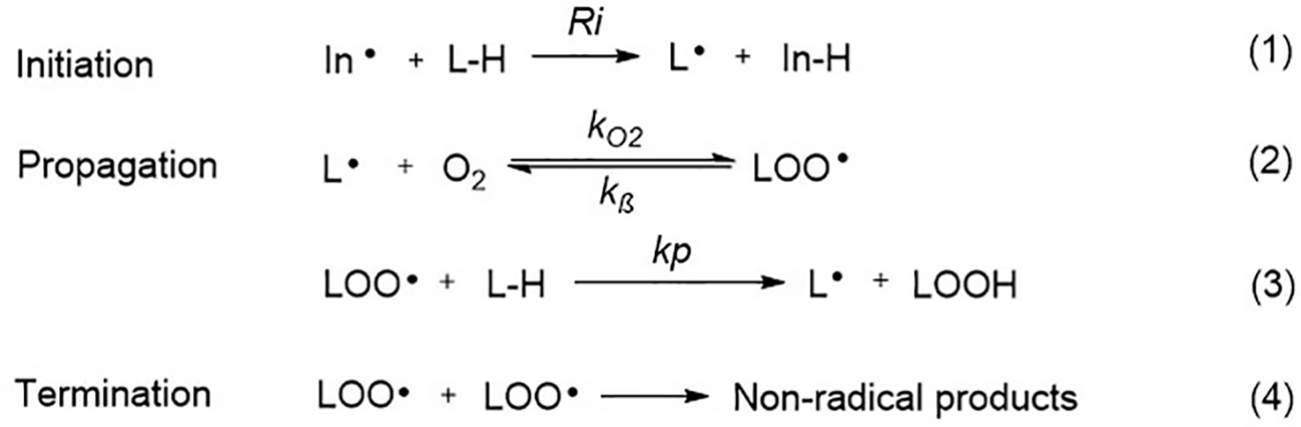	HT-1080 cell BJeLR cell Calu-1 cell
PE (Yang et al., 2014)	System x_c_^-^ and VDAC2/3	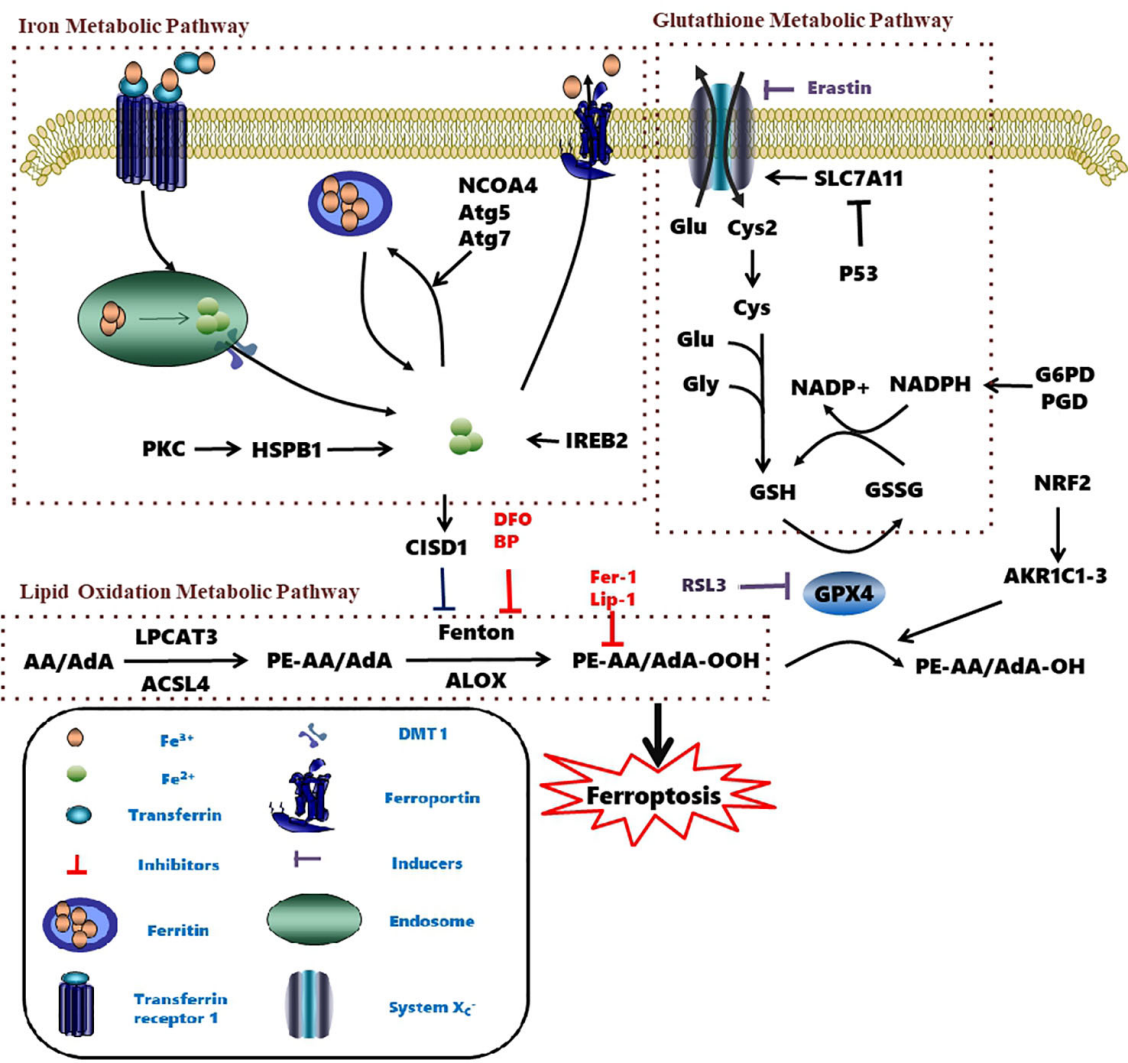	HT-1080 cell BJeLR cell DRD cell
IKE (Larraufie et al., 2015)	System x_c_^-^	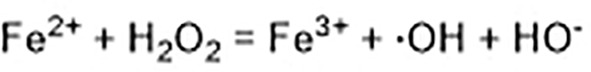	CCF-STTG1 cell HT-1080 cell BJeH cell BJeHLT cell DRD cell
Sorafenib (Louandre et al., 2013; Louandre et al., 2015)	System x_c_^-^ mitochondria ROS	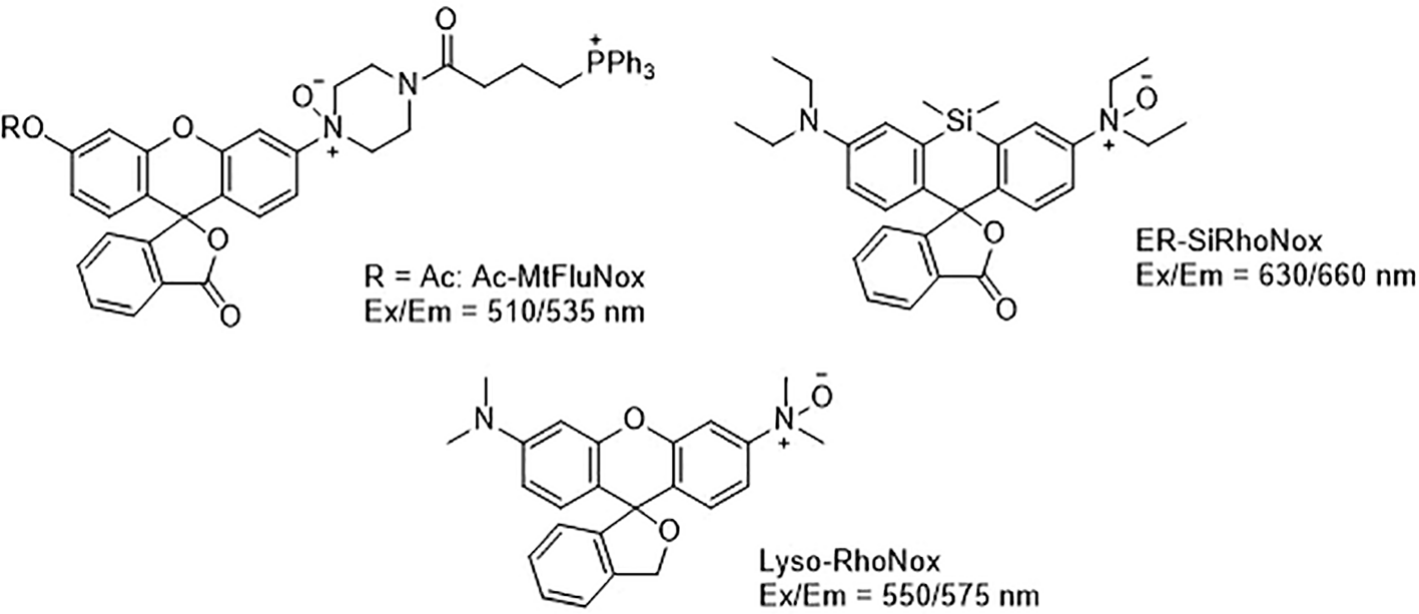	HCC cell
Sulfasalazine (Gout et al., 2001)	System x_c_^-^	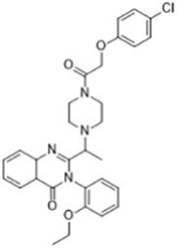	Nb2-SFJCD1 cell Nb2-U17 rats
Lanperisone (Shaw et al., 2011)	System x_c_^-^	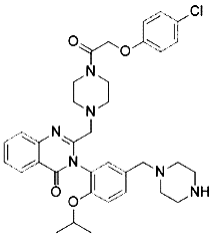	MEFs cell Mox2-Cre mice
RSL3 (Yang et al., 2014)	GPX4	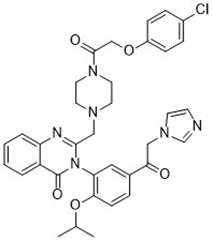	HT-1080 cell BJeLR cell BJeHLT cell 4 BJ cell
RSL5 (Yang and Stockwell, 2008)	VDAC2/3	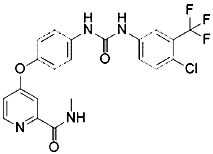	BJeH cell BJeHLT cell BJeLR cell
BSO (Yang et al., 2014)	GSH depletion	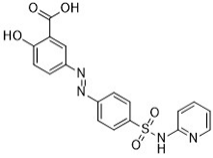	BJeLR cell BJeH cell BJeHLT cell DRD cell
DPI2 (Yang et al., 2014) (class I FINs)	GSH depletion	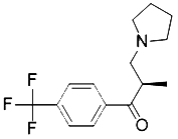	BJeLR cell
DPI7 (Yang et al., 2014) (class II FINs)	GPX4	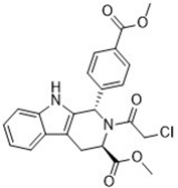	BJeLR cell
Acetaminophen (Lorincz et al., 2015)	GSH depletion	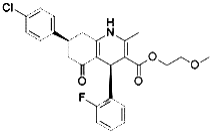	HepG2 cell NMRI mice
Artesunate (Eling et al., 2015)	GSH depletion	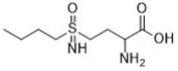	Panc1 cell COLO357 cell AsPC-1 cell BxPC-3 cell 293T cell HPDE cell PDAC cell
FIN56 (Shimada et al., 2016b)	Promote the degradation of GPX4 Reduce the abundance of CoQ_10_	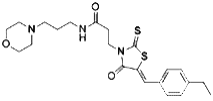	BJeLR cell DRD cell BJeHLT cell BJeH cell HT-1080 cell 143B cell Calu-1 cell
FINO_2_ (Gaschler et al., 2018a)	Oxidize ferrous iron Inactivate GPX4 indirectly	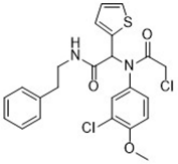	HT-1080 cell BJ-5ta cell BJeLR cell CAKI-1 cell
Silica-based nanoparticles (Kim et al., 2016)	GSH depletion and increase iron		M21 cell HT-1080 cell 786-O cell
Cisplatin (Yamaguchi et al., 2013)	GSH depletion	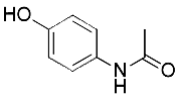	H1299 cell A549 cell PC9 cell

Erastin and RSL3 are the first ferroptosis inducers identified using high-throughput screening of small molecule libraries (Dolma et al., 2003; Yang and Stockwell, 2008). Erastin binds directly to mitochondrial voltage-dependent anion channel 2 (VDAC2) and causes ROS to be produced by NADPH-dependent pathway of mitochondrial damage (Yagoda et al., 2007). RNA interference mediates knockdown of VDAC2 or VDAC3 and leads to resistance to erastin-induced ferroptosis (Yagoda et al., 2007). In some tumor cells expressing active mutations, erastin induces cell death *via* the RAS-RAF-MEK pathway. In addition, erastin directly inhibits the activity of system x_c_^-^, resulting in the reduction in GSH production and inhibition of GPX4 activity, leading to accelerated production of ROS and ferroptosis (Dixon et al., 2012). Piperazine erastin (PE), a derivative of erastin, has better solubility and stability than erastin *in vivo*. It inhibits the proliferation of HT1080 cells by inducing ferroptosis (Yang et al., 2014). The carbonyl-containing erastin analog imidazole ketone erastin (IKE) has greater water solubility and metabolic capacity. It has been proved to be a more effective inducer of ferroptosis (Larraufie et al., 2015). It was also found that heme oxygenase-1 (HO-1) accelerates the occurrence of ferroptosis initiated by erastin through supplementing intracellular iron and producing ROS (Kwon et al., 2015). Sulfasalazine, a widely used molecule that treats chronic inflammation in the intestines, joints, and retina, can also inhibit system x_c_^-^ and induce ferroptosis while inhibiting the NF-ĸB signaling pathway (Gout et al., 2001). Similarly, sorafenib inhibits system x_c_^-^ and induces ferroptosis by non-kinase targets, enhanced toxicity to hepatocellular carcinoma (HCC) cells, and promoted persistent tumor regression (Louandre et al., 2013; Louandre et al., 2015).

Cell death induced by RSL3 and RSL5 shares a common feature with erastin-mediated ferroptosis. Similar to erastin, the initiation of ferroptosis induced by RSL3 and RSL5 is also dependent on the accumulation of ROS (Yang and Stockwell, 2008). The difference is that RSL3 is a direct inhibitor of GPX4, but not of system x_c_^-^ (Yang et al., 2014). RSL3 inactivates GPX4 and impedes the degradation of LOOH (Yang et al., 2014). On the other hand, RSL5 acts directly with VDAC2/3 to produce ROS (Yang and Stockwell, 2008).

The stimulant buthionine sulfoximine (BSO) irreversibly inhibits the activity of γ-glutamate cysteine synthetase, which is a rate-limiting enzyme in GSH synthesis in RAS mutant cells. It reduces the synthesis of GSH and further decreases the activity of GPX4 (Yang et al., 2014). Small molecule inducer FINs (**Table 2**) can be divided into two categories according to their mechanism of action. The first type of FIN, like DPI2, inhibits the activity of GPX4 by depleting GSH; the second type of FIN, such as DPI7, directly inhibits the activity of GPX4 and produces lipid ROS (Yang et al., 2014). Studies show that acetaminophen (Lorincz et al., 2015) and artesunate (Eling et al., 2015) kill cancer cells, not only by autophagy and apoptosis, but also by ferroptosis. In addition, cisplatin increases the level of intracellular ROS in the treatment of tumors to induce specific morphological changes in ferroptosis in cancer cells (Yamaguchi et al., 2013).

The recent discovery of a novel mechanism by which FIN56 and FINO2 trigger cellular death provides new insights on the regulation of ferroptosis (Shimada et al., 2016b; Gaschler et al., 2018a). FINO2 promotes lipid peroxidation by inducing iron oxide and indirectly inactivating GPX4 (Gaschler et al., 2018a). FIN56-induced cell death is accompanied by lipid ROS production and can be reversed by vitamin E and iron chelator, demonstrating that they are potent inducers of ferroptosis (Shimada et al., 2016b). Unlike the previously described pathways, FIN56 stimulates ferroptosis by consuming GPX4 proteins and blocking lipophilic antioxidants, such as coenzyme Q10 (Shimada et al., 2016b).

Many traditional Chinese medicinal natural products provide significant health benefits in the prevention and/or treatment of diseases. Traditional Chinese herbal extracts such as artemisinin (from Artemisia annua) initiate ferroptosis during the process of killing cancer cells (Eling et al., 2015). It induces ferroptosis in tumor cells by increasing ROS levels, decreases GSH levels, interferes with iron metabolism, and increases Fe^2+^ concentration (Eling et al., 2015). However, its mechanism of action related to signaling pathways has not been elucidated entirely. In 2018, Liu and his collaborators discovered a novel antitumor compound optimized from natural saponin Albiziabioside A. It induced ferroptosis as a p53 activator through the mitochondrial pathway (Wei et al., 2018).

In summary, ferroptosis can be triggered by blocking system x_c_^-^ with exogenous small molecules, interfering with GPX4, disrupting lipid metabolism balance, and iron homeostasis. Targeted therapy may be achieved by stimulating ferroptosis based on physiological differences between cancer cells and normal cells. Moreover, the rapid development of nanotechnology also provides new directions for the discovery of ferroptosis inducers.

## Inhibitors of Ferroptosis

To study the potential role of ferroptosis *in vivo*, effective and specific small molecular ferroptosis inhibitors have been identified using either high-through put screening or by designing compounds based on the structure-activity relationship. Most ferroptosis inhibitors can be classified into lipophilic radical-trapping antioxidants (e.g. Ferrostatin-1, α-tocopherol), iron chelators (e.g. deferoxamine), and deuterated polyunsaturated fatty acid phospholipids (PUFA-PLs) (Kagan et al., 2017). They have been approved to prevent ferroptosis and regulate normal function of intracellular iron metabolism, increase GSH levels, activate GPX4, or directly inhibit lipid peroxidation (**Table 3**).

**Table 3:** Inhibitors of Ferroptosis.

Compound	Target	Chemical structures	Model
Ferrostatin-1 (Skouta et al., 2014)	ROS from lipid peroxidation	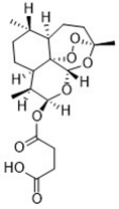	HT-1080 cell
SRS 11-92 (Skouta et al., 2014)	ROS from lipid peroxidation	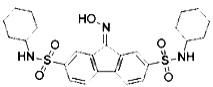	HT-1080 cell
SRS 16-86 (Linkermann et al., 2014)	ROS from lipid peroxidation	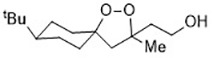	C57BL/6 mice FADD fl/fl mice RIPK3- deficient mice
Liproxstatin-1 (Friedmann Angeli et al., 2014)	ROS from lipid peroxidation	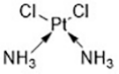	HRPTEpiCs cell HK-2 cell
α-tocopherol (Burton and Ingold, 2002)	Oxidative pathway	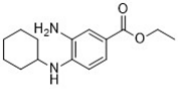	Pfa1 cell
2,6-di-tert-buyl-4-methylphenol (BHT) (Lucarini et al., 1996)	Oxidative pathway	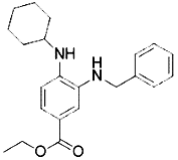	HT-1080 cell
β-carotene (Yagoda et al., 2007)	Oxidative pathway	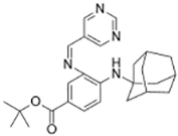	HT-1080 cell BJeLR cell Calu-1 cell
N-acetylcysteine (Dixon et al., 2012) (NAC)	Oxidative pathway	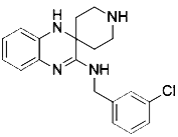	BJeH cell BJeHLT cell BJeLR cell HT-1080 cell DRD cell, MEFs cell
Ammonium chloride (Torii et al., 2016)	Regulation of iron equilibria and ROS generation	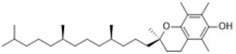	HT-1080 cell Calu-1 cell
Baf A1 (Torii et al., 2016)	Regulation of iron equilibria and ROS generation	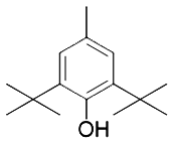	HT-1080 cell Calu-1 cell
PepA-Me (Torii et al., 2016)	Regulation of iron equilibria and ROS generation		HT-1080 cell Calu-1 cell
Deferoxamine (DFO) (Dixon et al., 2012)	Intracellular iron	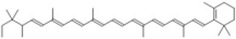	BJeH cell BJeHLT cell BJeLR cell HT-1080 cell DRD cell, MEFs cell
Deferoxamine mesylate (Yang and Stockwell, 2008)	Intracellular iron	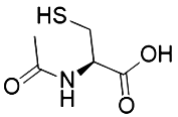	BJeHLT cell HT1080 cell MIA PaCa-2 cell BJeH cell, A549 cell BJeLR cell, Calu-1 cell
2,2'-bipyridyl (Dixon et al., 2012)	Intracellular iron	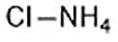	BJeH cell BJeHLT cell BJeLR cell HT-1080 cell DRD cell, MEFs cell
Ciclopirox olamine (Dixon et al., 2012)	Intracellular iron	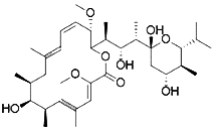	BJeH cell BJeHLT cell BJeLR cell HT-1080 cell DRD cell, MEFs cell
Zileuton (Yang et al., 2016)	Lipoxygenases	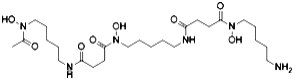	BJeH cell BJeHLT cell BJeLR cell HT-1080 cell
NDGA (Probst et al., 2017)	Lipoxygenases	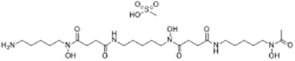	acute lymphoblastic leukemia cells
PD146176 (Yang et al., 2016)	Lipoxygenases	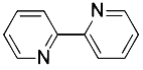	BJeH cell BJeHLT cell BJeLR cell HT-1080 cell
CDC (Yang et al., 2016)	Lipoxygenases	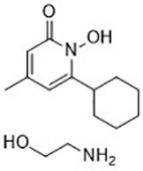	BJeH cell BJeHLT cell BJeLR cell HT-1080 cell
AA-861 (Yang et al., 2016)	Lipoxygenases	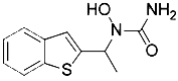	BJeH cell BJeHLT cell BJeLR cell HT-1080 cell
BW A4C (Angeli et al., 2017)	Lipoxygenases	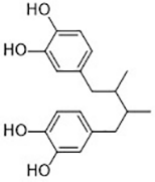	MEFs cell
Baicalein (Yang et al., 2016)	Lipoxygenases	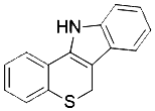	PANC1 cell BxPc3 cell
XJB-5-131 (Krainz et al., 2016)	Nitroxide antioxidant (Oxidative pathway)	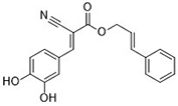	HT-1080 cell BJeLR cell panc-1 cell
Cycloheximide (Dixon et al., 2012)	Protein synthesis	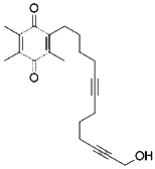	BJeH cell BJeHLT cell BJeLR cell HT-1080 cell DRD cell, MEFs cell
Diarylamine (Shah et al., 2017)	Radical-trapping antioxidant	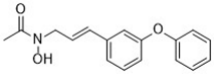	HepG2 cell Pfa1 cell
Phenoxazine (Shah et al., 2017)	Radical-trapping antioxidant	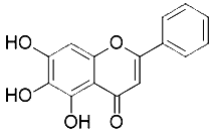	HepG2 cell Pfa1 cell
Phenothiazine (Shah et al., 2017)	Radical-trapping antioxidant	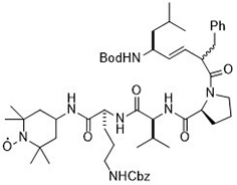	HepG2 cell Pfa1 cell
Tetrahydronapthyridinols (Angeli et al., 2017) (THNs)	Radical-trapping antioxidant	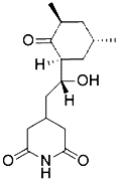	HEK293 cell MEFs cell
PMC (Shah et al., 2018)	Radical-trapping antioxidant	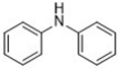	MEFs cell
TEMPO (Griesser et al., 2018)	Radical–trapping antioxidant	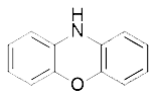	MEFs cell
Rosiglitazone (Angeli et al., 2017)	ACSL4	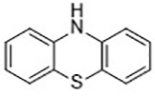	Caco-2 cell C57BL/6 mice
Pioglitazone (Angeli et al., 2017)	ACSL4	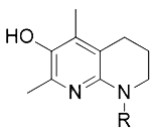	HepG2 cell Hep3B cell
Troglitazone (Angeli et al., 2017)	ACSL4	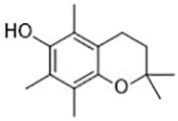	Human breast cancer cell lines SK-BR-3 cell

Ferrostatins and liproxstatins act as free radical-trapping antioxidants to inhibit lipid peroxidation associated with ferroptosis (Yang and Stockwell, 2016). The first generation of ferrostatins Ferrostatin-1 (Fer-1 in **Table 3**) inhibits erastin and RSL3-induced ferroptosis in HT1080 cells. Their activity is dependent on the aromatic amines, which specifically inhibit lipid peroxidation (Yang and Stockwell, 2016). The second (SRS11-92) and third generation (SRS16-86) ferropstatins show significant enhancement cellular metabolism and damage prevention compared to the first generation Fer-1 (Ran et al., 2015). Fer-1 is currently considered as a probe for studying ferroptosis in different environments and as a basic potential drug molecule against lipid peroxidation-mediated tissue damage (Kabiraj et al., 2015).

Liproxstatin-1 (Lip-1) contains amide and sulfonamide subunits. It has good stability and drug absorption distribution *in vivo* (Hofmans et al., 2016). It can inhibit ferroptosis at low nano molar dose, but it does not interfere with other typical cell death patterns (Hofmans et al., 2016). Similar to ferrostatins and liproxstatins, the antioxidants α-tocopherol (Burton and Ingold, 2002), BHT (Lucarini et al., 1996), N-acetylcystein (NAC) (Dixon et al., 2012), *etc.*, can block ferroptosis by inhibiting the lipid peroxidation pathway.

Free polyunsaturated fatty acids are the substrates of LOXs, which mediate ferroptotic peroxidation (Kuhn et al., 2015). Studies show that inhibitors of ferroptosis, such as tocopherols (Khanna et al., 2003) and flavonoids (Xie et al., 2016b), can also inhibit LOXs activity in some cases. However, not all lipoxygenase inhibitors inhibit ferroptosis. Effective lipoxygenase inhibitors, such as CDC and zileuton, are free radical-trapping antioxidants (Yang et al., 2016). For example, 5-lipoxygenase (5-LOX) inhibitor zileuton inhibits glutamate toxicity and ferroptosis by inhibiting cytoplasmic ROS production in HT22 cells, thereby exerting neuroprotective effects (Liu et al., 2015).

Ferroptosis can also be inhibited by iron chelators such as deferoxamine (DFO), deferoxamine mesylate, and 2, 2'-bipyridyl (Dixon et al., 2012) since they prevent the initiation of lipid peroxidation by inhibiting Fenton chemistry. Natural products have also been screened out for ferroptosis inhibitors. In 2016, Tang's lab found out baicalein was an effective ferroptosis inhibitor in pancreatic cancer cells (Xie et al., 2016b) functioned by reducing ROS, regulating iron homeostasis, chelating Fe^2+^, and protecting GPx4 (Xie et al., 2016b).

Moreover, inhibition of heat shock factor-1 (HSF-1) dependent heat shock protein beta 1 (HSPB1) expression can also inhibit erastin-induced ferroptosis (Sun et al., 2015). In addition, Nrf2 plays an important role in the anti-ferroptotic process in liver cancer cells. Upregulation of Nrf2 initiates transcription of antioxidant protein genes and iron metabolism protein genes, thereby inhibiting ferroptosis (Sun et al., 2016). Fms-like tyrosine kinase 3 (Flt3) and phosphoinositide 3-kinase α (PI3Kα) inhibits neuronal ferroptotic cell death, particularly in cortical neurons, providing neuroprotection (Kang et al., 2014).

Other than the screened compounds, research has also focused on designing synthetic ferroptosis inhibitors in order to provide potential health benefits to prevent and/or ease the symptom of diseases that are related to ferroptosis. Several series of effective ferroptosis inhibitors have been developed in recent years (Zilka et al., 2017). Radical-trapping antioxidants, including tetrahydronapthyridinols (THNs), phenoxazine, phenothiazine, and diarylamine (**Table 3**), have been shown to effectively inhibit ferroptosis in cellular models, with some exhibiting better activity than Fer-1 and Lip-1. Recently, we have designed and synthesized a series of chalcone derivatives to inhibit amyloid-β aggregation and ferroptosis at the same time in cellular models as a potential preventive and/or therapeutic agent to Alzheimer's disease (Cong et al., 2019). Together, these findings suggest the possibility for introducing multi-functional ferroptosis inhibitors to prevent and/or treat diseases that are closely associated with ferroptosis.

## Potential Role of the Initiators and Inhibitors of Ferroptosis in the Treatment of Various Diseases

### Neurodegenerative Diseases

Neurodegenerative diseases, such as Alzheimer's disease (AD) and Parkinson's disease (PD), are known to be associated with dysregulation of iron homeostasis and excessive ROS in the brain. Before the concept of ferroptosis, neurodegenerative diseases were thought to be caused by apoptosis (Ward et al., 2014). With the definition of ferroptosis in 2012 and iron-dependent oxidative stress as a significant marker of cellular ferroptosis, there is an increasing amount of research supporting the idea that ferroptosis is inextricably linked to neurodegenerative diseases.

Alzheimer's disease is one of the most common causes of dementia in aging individuals. It is characterized by progressive memory impairment and cognitive dysfunction. The main pathological features of AD are extracellular β-amyloid (Aβ) deposition and neurofibrillary tangles caused by abnormal phosphorylation of intracellular Tau protein. There is evidence supporting that oxidative stress and iron metabolism disorder are associated with the progression of AD (Masaldan et al., 2019).

Ferroptosis is characterized by an accumulation of lipid peroxidation and dysregulation of iron, which are precisely the hallmarks of Alzheimer's disease (Pratico and Sung, 2004; Castellani et al., 2007). Therefore, regulating ferroptosis has become a new direction for the potential treatment of Alzheimer's disease. Iron chelators also prevent the development of AD by maintaining levels of hypoxia inducible factor-1 alpha (HIF-1α) in the nerve and inhibiting neuronal death, which provides a novel neuroprotective mechanism against AD (Ashok et al., 2017). In addition to the treatment of AD by a single iron chelator, multi-target drugs by chelating Fe (II) combined with scavenging free radicals may also be effective. As a multifunctional non-toxic and brain-permeable iron chelator, M30 not only attenuates Tau phosphorylation but also activates the HIF-1α signaling pathway, showing great potential in the prevention and treatment of AD (Kupershmidt et al., 2012). Alpha-Lipoic acid not only regulates the redistribution of iron *via* iron chelation, but also acts as a direct free radical scavenger and an indirect antioxidant which can inhibit ferroptosis to alleviate the progression of AD (Zhang et al., 2018a). According to our understanding, the radical-trapping antioxidant α-tocopherol and the iron chelator DFO entered clinical testing to treat AD before they were discovered as inhibitors of ferroptosis (Mclachlan et al., 1991; Dysken et al., 2014). Clinical trials proved that patients with mild to moderate AD who received 2000 IU/day α-tocopherol showed a slower decline in cognitive function compared to the placebo group (Dysken et al., 2014).

Parkinson's disease is the second most common neurodegenerative disease and it is characterized by the loss of dopaminergic neurons in the substantia nigra and the formation of cytoplasmic eosinophilic inclusion bodies, *i.e.*, Lewy bodies (Hornykiewicz, 2008). It is currently believed that lipid peroxidation of dopaminergic neurons in the substantia nigra pars compacta is important in the pathogenesis of PD (Burbulla et al., 2017). Some pathological features found in PD patients are elevated levels of free iron in the substantia nigra neurons, lipid peroxide production, and accumulation of ROS, are closely related to ferroptosis. In mammalian models, several studies have shown that iron chelators can protect against neuronal damage in PD (Kaur et al., 2003; Ayton et al., 2013; Lei et al., 2015). For example, in 2014, clinical studies of the deferiprone (DFP), an iron chelator, has shown that iron-removing treatment can alleviate the motor symptoms of early PD patients by reducing iron levels in patients (Devos et al., 2014). Iron chelator VK28, or its derivative, M30, which can penetrate the blood-brain barrier, provide significant neuroprotective effects in PD mouse models (Avramovich-Tirosh et al., 2010). In addition, genetic studies of PD have shown that PD marker α-synuclein, encoded by the SNCA gene, connect strongly with ferrous and ferric ions (Peng et al., 2010; Febbraro et al., 2012). These two forms of iron ions have been shown to accelerate the aggregation of α-syn *in vitro*, and the iron chelators can significantly inhibit this phenomenon (Hashimoto et al., 1999; Davies et al., 2011). For instance, deferoxamine (DFO) can be used to treat HEK293 cells, confirming that iron deficiency could inhibit the expression of α-synuclein and prevent PD-like changes in cells (Febbraro et al., 2012).

Recently, Huntington's disease (HD), a hereditary neurodegenerative disorder, has also been shown to be inextricably linked to ferroptosis. Similar to Alzheimer's and Parkinson's diseases, Huntington's disease also associates with abnormal levels in lipid peroxidation, GSH metabolism, and iron accumulation (Paul et al., 2014). Increasing lipid peroxidation was detected in cortical striatal brain sections (Skouta et al., 2014) and cerebrospinal fluid (Reddy and Shirendeb, 2012) of the mN90Q73 HD mouse model. 3-nitropropionic acid (3-NP)-induced HD mice display a decrease in GSH and GSH-S-transferase in the striatum, cortex, and hippocampus (Kumar et al., 2010). Moreover, increasing iron supplementation reduces the striatum volume and contributes to neurodegeneration (Van Bergen et al., 2016). In contrast, intracerebroventricular administration of deferoxamine (DFO) improves striatal pathology and motor phenotype in R6/2HD mice (Chen et al., 2013).

Cell stress response to ROS includes the activation of pro-survival pathways as well as the production of molecules endowed with antioxidant and anti-apoptotic activities, which is under the control of protective genes called vitagenes (Calabrese et al., 2009). Vitagene network includes members of the heat shock protein (HSP) family, such as heme oxygenase-1 (HO-1), Hsp70, sirtuins (Sirt-1), and thioredoxin/thioredoxin reductase (Trx/TrxR) (Calabrese et al., 2009). Heat shock factors (HSFs) are the master transcriptional factors that regulate the inducible synthesis of these HSPs during stress (Wu, 1995). In addition to HSF, some of the vitagenes are also upregulated as part of the phase 2 responses, a cytoprotective response that protects against various electrophiles and oxidants (Calabrese et al., 2007). Vitagene network including heme oxygenase 1, thioredoxin, and thioredoxin reductase can be upregulated by the transcription factor Nrf2 (Calabrese et al., 2010b). It has been found that GPX4, heat shock protein beta-1(HSPB1), and Nrf2 function as negative regulators of ferroptosis by limiting ROS production and reducing cellular iron uptake, respectively (Xie et al., 2016a). The protection effect of HSPs in ferroptosis has been elucidated in the past few years. In 2015, Tang's group found that inhibition of HSF-1-dependent HSPB1 expression increased ferroptosis, whereas overexpression of HSPB1 inhibited erastin-induced ferroptosis (Sun et al., 2015). They also found that the PKC-mediated HSPB1 phosphorylation in Hela cells was required for conferring resistance to erastin-induced ferroptosis, possibly through regulating iron-mediated lipid ROS production (Sun et al., 2015). In 2017, Tang's group showed that heat shock 70-kDa protein 5 (HSPA5) negatively regulated ferroptosis in human pancreatic ductal adenocarcinoma (PDAC) cells through the HSPA5-GPX4 pathway (Zhu et al., 2017). Mechanistically, activating transcription factor 4 (ATF4) resulted in the induction of HSPA5, which in turn bound glutathione peroxidase 4 (GPX4) and protected against GPX4 protein degradation and subsequent lipid peroxidation (Zhu et al., 2017). Due to the known neuron protection effect of HSPs *in vitro*, it provides a potential therapeutic strategy for acute injury in the nervous system.

In conclusion, current studies on the role of ferroptosis in neurodegenerative diseases mainly concentrate on studying whether ferroptosis inhibitors could slow disease progression, and mostly use animal models. Most of the experimental studies in animals have shown that effective inhibition of ferroptosis provided potential treatment. However, most clinical trials on administering iron chelators and antioxidants showed only moderate treatment effect. These results lead us to think that iron chelators and antioxidants are not sufficient to provide effective treatment. Potential molecules that regulate ferroptosis through other signaling pathways have yet to be further explored for their potential to treat neurodegenerative diseases and could provide better treatment.

### Cancer

Most cancer cells exhibit elevated levels of ROS (Hassannia et al., 2019). They rely on the level of glutathione to maintain their survival and proliferation (Di Meco et al., 2017). Raising the level of ROS to a cytotoxic level can eliminate cancer cells. Endogenous cysteine produced under elevated ROS levels is not sufficient to synthesize sufficient glutathione. Therefore, extracellular cysteines need to be obtained by the reverse transporter system x_c_^-^ (Dixon et al., 2014). Erastin (Dixon et al., 2012), sorafenib (Louandre et al., 2013), and sulfasalazine (Gout et al., 2001) have been explored as inhibitors of system x_c_^-^ to stimulate ferroptosis in cancer cells.

Ferroptosis was first discovered in tumor cells when studying RAS mutations. In 2014, Stockwell's group (Yang et al., 2014) studied the possibility of using ferroptosis inducers for RAS mutant cancer cells by measuring erastin from 117 cancer cell lines from different tissues. Results showed that kidney cancer cells (RCCs) and leukemia (DLBCL) are more sensitive to erastin than other cancer cells, like those found in lung and ovarian cancers. They also demonstrated that RAS mutations are not associated with the efficacy of erastin. In addition, erastin can enhance the efficacy of chemotherapy drugs, such as temozolomide (Chen et al., 2015) and cisplatin (Yamaguchi et al., 2013), to treat specific cancer cells. The analogs of erastin, piperazine erastin (PE) (Yang et al., 2014), and imidazole ketone erastin (IKE) (Larraufie et al., 2015), have also been used as *in vivo* probes for tumor susceptibility to ferroptosis.

In 2015, Jian and colleagues (2015) elucidated a new role for P53 in the mediation of tumor suppression through ferroptosis. The authors demonstrated that P53 inhibits the uptake of cystine through the inhibition of SLC7A11 gene expression, resulting in ferroptosis. This conclusion also indicates that P53 wild-type tumors can be treated with ferroptosis inducers that inhibit system x_c_^-^. Interestingly, SLC7A11 gene-deficient mice develop normally and healthy (Sato et al., 2005), suggesting that system x_c_^-^ targeted drugs with high cancer cell selectivity have few side effects in preclinical studies.

In addition, it has been found that the ACSL4 enzyme is preferentially expressed in a subset of triple-negative breast cancer cells (TNBC) and that expression of the ACSL4 enzyme is closely related to stimulating ferroptosis (Doll et al., 2017). Since triple-negative breast cancer is difficult to control, ferroptosis initiation introduces a new method for the treatment of cancer cells with ACSL4 expression (Doll et al., 2017).

In recent years, with the rapid development of nanotechnology, studies have shown that tumor xenografts in mice that use high-dose multiple intravenous injections of polyethylene nanoparticles coated with polyethylene glycol exhibit a slower growth of cancer and even signs of regression (Szwed et al., 2019). However, this phenomenon is reversed by ferroptosis inhibitor liprostatin-1 (Kim et al., 2016). This suggests that ferroptosis could have great potential for targeted cancer treatment through ultra-small silica nanoparticles.

Currently, FDA-approved drugs sorafenib (Louandre et al., 2013), sulfasalazine (Gout et al., 2001), artesunate (Eling et al., 2015), and lanperisone (Shaw et al., 2011) have been shown to induce ferroptosis in certain cancer cells. Among them, sorafenib-induced cellular ferroptosis had two different mechanisms: (1) inhibition of system x_c_^-^ mediated cystine input and triggering endoplasmic reticulum stress (Dixon et al., 2014); (2) reduction of Rb protein, which is best known for its regulatory role in cell proliferation and its key role at the G1/S checkpoint accompanied by increased ROS in mitochondria (Louandre et al., 2015). However, further research is necessary to determine whether endoplasmic reticulum stress is the key initiator of cancer cell death after treatment with sorafenib. Further studies are needed to elucidate the mechanism of Rb protein production.

### Ischemia Reperfusion Injury

Ferroptosis inhibitors have been used to treat a variety of kidney injuries, such as ischemia-reperfusion and oxalic acid-induced kidney damage (Gascon et al., 2016), rhabdomyolysis (Bosch et al., 2009), and acute renal failure (ARF) (Bosch et al., 2009). Ferroptosis inhibitor Fer-1 prevents cell death in an *in vitro* model of rhabdomyolysis-induced acute kidney injury (Skouta et al., 2014). In an *in vivo* model of renal ischemia-reperfusion injury, SRS16-86, a third generation ferrostatin with increased plasma and metabolic stability, protected renal function and prolonged survival after ischemia-reperfusion injury (Linkermann et al., 2014). Ferroptosis inhibitor Lip-1 can rescue acute renal failure and prolong life in mice due to GPX4 deletion (Friedmann Angeli et al., 2014). In addition, thiazolidinediones (TZDs) inhibit acyl-CoA synthase 4 and partially reduce the mortality of induced GPX4 knockout mice (Doll et al., 2017). These results reinforce the sensitivity of kidney tissue to ferroptosis and demonstrate the value of ferroptosis inhibitors in the treatment of renal damage (Sancho-Martinez et al., 2015).

When the isolated cardiac ischemia-reperfusion model of wild-type mice is treated with glutaminolysis inhibitor compound 968 and iron chelator DFO, the cardiac function is significantly enhanced when compared to the control group (Gao et al., 2015). This indicates that heart damage caused by ischemia-reperfusion can be reduced by inhibiting glutaminolysis, which is the essential component in ferroptosis. Fang and colleagues (2019) used a variety of cell death inhibitor treatments and cell death pathway-related knockout mouse models to find that only the ferroptosis-specific inhibitor Fer-1 could significantly reduce the cardiotoxicity caused by DOX, an anticancer drug. It was revealed that ferroptosis was involved in the mechanism of myocardial injury. The researchers also found the presence of ferroptosis in a mouse model of myocardial ischemia-reperfusion injury. The administration of ferroptosis inhibitors to block ferroptosis could significantly reduce acute and chronic heart damage caused by ischemia-reperfusion. These results provide new ideas and strategies for clinical heart diseases such as myocardial infarction.

Excessive acetaminophen is the most common cause of acute liver failure. Acetaminophen has been shown to induce ferroptosis in primary hepatocytes, while ferroptosis inhibitors such as Fer-1 inhibit acetaminophen-induced cell death (Lorincz et al., 2015). Moreover, Lip-1 repairs liver damage caused by ischemia-reperfusion (Friedmann Angeli et al., 2014). Collectively, these findings show the importance of ferroptosis in ischemia reperfusion injury and support the potential therapeutic application of ferroptosis inhibitors that target pathways involved in ferroptosis execution.

### Other Diseases

Excessive accumulation of iron ions causes lipid peroxidation and tissue damage, leading to atherosclerosis and diabetes (Wu and Chen, 2015). Studies have shown that iron overload in the heart caused myocardial dysfunction and metabolic damage that ultimately led to heart disease (Dixon et al., 2012). In GPX4-deficient T cells, the cell membrane rapidly accumulates lipid peroxides, which induces ferroptosis. Instead, inhibiting ferroptosis promotes the survival and expansion of T cells and protects the immune function of T cells (Matsushita et al., 2015). Research also shows that ferroptosis participates in keratinocyte death due to GSH loss, and high doses of vitamin E can inhibit ferroptosis of skin keratinocytes and reduce skin damage (Wu and Chen, 2015). Recent studies show that the decreased expression of frataxin, a key protein of Friedreich's ataxia (FRDA), characterized by puberty onset, loss of tendon reflexes, and deep sensory loss, is associated with mitochondrial dysfunction, mitochondrial iron accumulation, and increased oxidative stress. Ferroptosis inhibitor SRS11-92 reduces cell death caused by FRDA (Cotticelli et al., 2019).

For a long time, researchers believed that the secondary damage caused by intracranial hemorrhage was caused by the random spread of iron ions. However, the discovery of ferroptosis has led more researchers to wonder whether the damage caused by intracranial hemorrhage is the induction of ferroptosis in cells. Alim and colleagues (2019) not only validated in hemorrhagic stroke models that stroke induces ferroptosis to some extent, but also found that the expression of the GPX4 can be driven by delivering a single dose of selenium to the brain, thereby protecting neurons and improving the behavior of mice after stroke (Green, 2018; Ingold et al., 2018). These findings provide important guidance in the nutritional care and follow-up treatment of cerebral hemorrhage.

In summary, the survival of cells is an important part of the body's normal metabolism. It is clear that ferroptosis has an intimate relationship with pathological cell death. Effective alleviation or prevention of the progression of the disease or the clinical symptoms in mice or rat models can be achieved by administering ferroptosis inhibitors or inducers. Emerging evidence also suggests that ferroptosis initiation has a potential tumor inhibitory function, which could clear tumor cells that lack key nutrients in the environment and cells that are damaged by infection (Yang et al., 2014). In-depth study and clarification of the pathophysiological mechanism of ferroptosis in related diseases will provide new ideas for discovering potential drug targets and clinical prevention methods.

## Summary

Different from other cell death patterns induced by cytosolic or mitochondrial reactive oxygen species, ferroptosis is defined as a form of programmed cell death involving the accumulation of lipid hydroperoxides which can be suppressed by iron chelators and lipophilic antioxidants. It is characterized by the loss of activity of enzyme GPX4, which results in the accumulation of lethal lipid hydroperoxides. Topics on signaling pathways involved in ferroptosis, the role of ferroptosis inhibitors and initiators as well as their mechanism of action, the role of ferroptosis in disease, and the difference between ferroptosis and other cell death types involving excessive reactive oxygen species, have been widely studied in the past few years.

The difference between ferroptosis and other cell death pathways caused by excessive oxidative damage *in vivo* has not been elucidated yet. Recent studies indicate that ferroptosis shares a few common characteristics with several types of cell death pathways, like oxytosis (Neitemeier et al., 2017) and ferritinophagy (Zhang et al., 2018b), with a few differences in protein-signaling pathways. In cellular models, the morphology and characteristics of cells and mitochondria can be explored to provide evidence to distinguish ferroptosis from other types of cell death. However, it is challenging to do so in the diseased animal models. In addition, different cell death patterns might happen and contribute to the pathology of disease at the same time. For example, it is already known that both ferroptosis and necroptosis happen after ischemic injury (Linkermann et al., 2014) and both apoptosis and ferroptosis occur after traumatic brain damage (Raghupathi et al., 2000; Magtanong and Dixon, 2018). Therefore, detection of markers of one cell death type alone cannot indicate the lack of other cell death types in diseased model.

It is known that ROS in the biological system has hormesis feature, which means although excess ROS is harmful to keep the redox balance, small amounts of ROS, such as mitochondrial superoxide and hydrogen peroxide, play important roles in a range of cellular functions, and can also activate signaling pathways that promote cell survival and disease resistance (Calabrese et al., 2008; Mattson, 2008; Calabrese et al., 2010a). An example of cellular hormesis mediated by ROS is the study showing that oxidative stress can stimulate angiogenesis in the brain, a process that is very important in restoring blood flow to neurons after a stroke (Hougaard et al., 2013; Wei et al., 2013). To date, it is not yet clear whether ferroptosis has hormesis feature. However, some known ferroptosis inhibitors, such as the natural products curcumin and baicalein, have hormesis feature (Wang et al., 2018; Concetta Scuto et al., 2019). At low concentration, curcumin and baicalein inhibit ferroptosis in cellular models (Concetta Scuto et al., 2019; Li et al., 2019). However, at concentration above a threshold, they induced toxicities. Therefore, special precaution is necessary when applying ferroptosis inhibitors as potential protective agents.

In addition, the interpretation of cell-based data and animal-based data related to the link between ferroptosis and neurodegenerative diseases must also be carefully considered since the *in vitro* cellular experimental conditions are quite different from *in vivo* conditions. To date, there is no clinical trial using ferroptosis inhibitors or initiators to treat degenerative diseases (see www.clinicaltrials.gov for details). Further research on the mechanism through which lipid peroxidation induces ferroptotic cell death is necessary.

## Author Contributions

The original idea of this study was from BL. CH and YL reviewed the literature and contributed equally to the manuscript writing and editing. BL instructed the whole manuscript formation. RD contributed to the manuscript editing and proofreading. NI and WS contributed to the manuscript revising.

## Funding

The authors were supported by the grants from the Fund of Innovation Center of Radiation Application, China (KFZC2018040208), the National Natural Science Foundation of China (31971388), and the Beijing Institute of Technology Research Fund Program for Young Scholars.

## Conflict of Interest

The authors declare that the research was conducted in the absence of any commercial or financial relationships that could be construed as a potential conflict of interest.
